# Power Law Scaling in Human and Empty Room MEG Recordings

**DOI:** 10.1371/journal.pcbi.1004175

**Published:** 2015-05-08

**Authors:** Manfred G. Kitzbichler, Edward T. Bullmore

**Affiliations:** Behavioural & Clinical Neuroscience Institute, University of Cambridge, Cambridge, United Kingdom; John Radcliffe Hospital, United Kingdom

## Overview

Recently published results seemed to show power law scaling of phase synchronization, a characteristic that we have previously interpreted as indicative of critical dynamics, in “empty room” MEG recordings. Here we show that the results we previously reported on scaling of phase synchronization in human MEG data are not compromised by these apparently anomalous results. By extensively reanalysing the empty room MEG data using identical methods to those previously used for analysis of human MEG data, we demonstrate the validity of our prior results and explain the anomalous empty room results.

## Introduction

In 2009, we published a paper in *PLOS Computational Biology* [1] that described using a new, wavelet-based metric of phase synchronization in human MEG data. Specifically, we showed that this metric of phase synchronization, that we called the phase lock index (PLI), demonstrated power law scaling across all frequency intervals or wavelet scales from the low frequency delta band (1–2 Hz) to the high frequency gamma band (35–70 Hz). Based on these experimental results, and additional confirmatory data obtained from PLI measurements on time series generated by computational models of critical systems, we offered the interpretation that power law scaling of phase synchronization in human MEG recordings was compatible with the prior theory that human brain dynamics demonstrate self-organized criticality.

**Fig 1:**
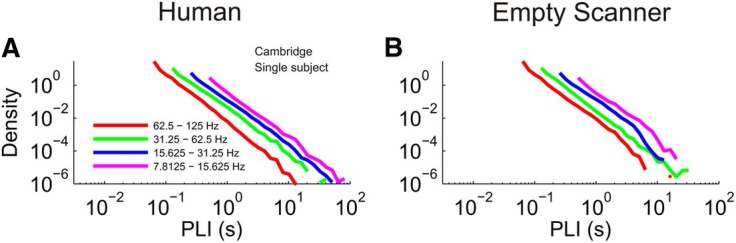
First two panels of Figure 9 from Shriki et al. [2]. These results seem to indicate that “PLI analysis yields similar results for human and empty scanner data.” The panels show the PLI distributions of a single human subject (A) and a single empty room recording (B), both at the Cambridge MEG facility.

More recently, in collaboration with a group at the National Institutes of Health (NIH), we published a paper in the *Journal of Neuroscience* [2] that described using a similar phase synchronization metric to explore scaling behaviour in MEG data recorded from normal human subjects and, crucially, in MEG data recorded with no human subject present, so-called “empty room” data. In Figure 9 of [2], we showed data indicating that phase synchronization appeared to demonstrate power law scaling even in empty room data; as reproduced here in Fig 1.

We originally judged this issue to be of minor concern, because, as shown in Figure 1 of [2], NIH empty scanner amplitude variance is about 1–2 orders of magnitude less than equivalent brain scans. This is most likely an underestimation given that data are Z-normalized and absolute amplitudes of empty scanner data should be lower than brain scans. Thus, we reasoned the contribution from “empty scanner” effects to PLI scaling in brain recordings should be insignificant. Nonetheless, the issue was addressed in the Discussion of [2], where we stated:

“because of the ambiguity of PLI for brain scans and empty scanner, additional steps such as amplitude comparisons need to be taken into account” (page 7089 of [2]).

The editors of *PLOS Computational Biology* were subsequently contacted by Dr. Farmer, who made the following point:

“In [2] and [1]…a power law relationship was discovered in a measure of MEG inter-areal synchronisation; the distribution of phase locking intervals (PLI). However, in [2] the authors also show that the same PLI power law measure cannot distinguish between human MEG and empty MEG scanner data, suggesting that the measure is vulnerable to artefact.”

We accept the principle of this criticism. If power law scaling can be demonstrated, by PLI or any other metric, in MEG data recorded from an empty room then this is conceptually problematic for interpretation of power law scaling in human MEG data as a marker of self-organized criticality, or any other complex biological dynamics.

To assess the extent to which this criticism actually applies to the results reported by [1], we have therefore undertaken extensive additional analyses of both the human and empty room MEG data reported by Shriki et al. [2].

## Synopsis of Methods Used by Kitzbichler et al. [1] and Shriki et al. [2]

We begin by clarifying exactly how the MEG data in Kitzbichler et al. [1] were collected, pre-processed, and analyzed for phase synchronization. As described in more detail in the original paper, these data were collected at the MRC Cognition & Brain Sciences Unit in Cambridge, United Kingdom, using an Elekta machine, comprising a magnetometer and two planar gradiometers, each at 102 locations. These data were pre-processed to mitigate line noise at 50 Hz and its harmonics by applying a narrow band stop filter, also called a “notch” filter. Phase synchronization was estimated by a wavelet-based estimator (PLI).

The paper by Shriki et al. [2] reported some of these Cambridge data, but used a different pre-processing pipeline, which did not include notch filtering of line noise, and used a different computational approach to estimate phase synchronization (see below for details). Shriki et al. also reported results from MEG data collected at the NIH, Bethesda, United States of America (as well as some data from Cambridge). The NIH MEG system is a CTF machine that comprises exclusively radial gradiometers at 275 locations around the skull. The pre-processing operations applied to these data did not include filtering to exclude line noise at 60 Hz and its harmonics. Phase synchronization was measured by Shriki et al. using a method that was similar, but not identical, to the wavelet-based method reported by Kitzbichler et al. To mitigate the algorithmic complexity and high computational cost of the wavelet-based PLI method used by Kitzbichler et al., Shriki et al. used ordinary band-pass filtering in conjunction with a Hilbert transform to construct an analytic signal. Also, the sliding window procedure used by Kitzbichler et al. to estimate a time-resolved measure of quasi-instantaneous coherence was simplified so that an existing compiled library could be used instead of the comparatively slower interpreted functions in R or Matlab.

Thus, it was clear, on close review of the methods used in the two papers, that the results were based on different pre-processing pipelines and somewhat different estimators of phase synchronization. In short, the two methods differed in detail and they were not exactly the same.

## Reanalysis of MEG data Reported by Shriki et al.

To investigate the impact of these differences in MEG pre-processing and estimation of phase synchronization, we reanalysed the Cambridge and NIMH data previously reported by Shriki et al. using the methods implemented by Kitzbichler et al. Specifically, we used a pre-processing pipeline that optionally included notch filtering to remove line noise, and we used the wavelet-based estimator of PLI as originally proposed.

**Fig 2:**
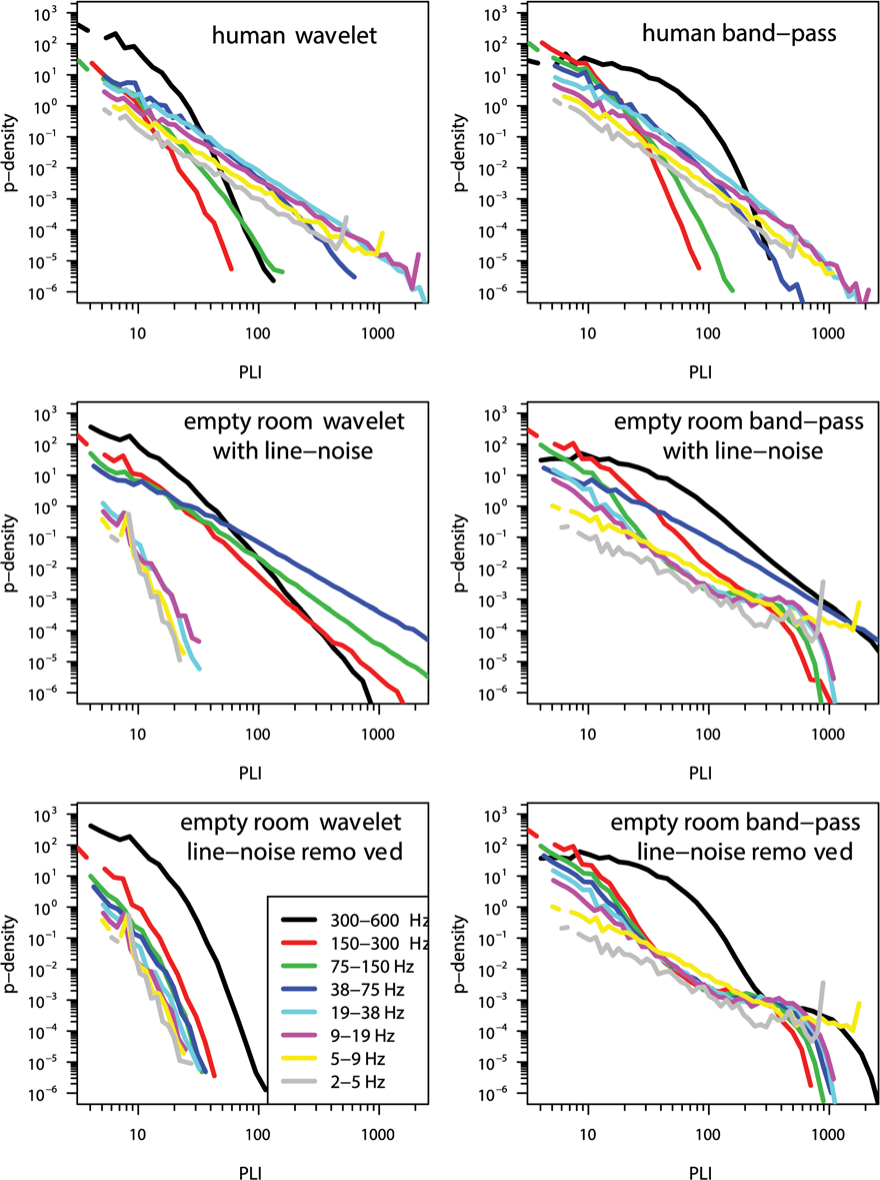
Reanalysis results. Original wavelet-based method of PLI analysis (left panels; from [1]) and simplified band-pass method of PLI analysis (right column; from [2]) applied to same data as in [2]. The top row shows human subject data, the middle row shows empty-room data with line noise included, and the bottom row shows empty room data with line-noise artefacts removed.

Some of the results of this reanalysis are summarised in Fig 2 (very similar results were obtained for all other datasets reported by Shriki et al.). First, we were able to reproduce the previous reports of power law scaling of synchronization metrics in data recorded from human subjects specifically at neurophysiological frequencies (2–75 Hz), and not at very high frequencies (>75 Hz). However, when we reanalysed the empty room data using the wavelet-based PLI estimator, there was no evidence for power law scaling at neurophysiological frequencies. Moreover, when we pre-processed the empty room data to remove line noise, and then estimated synchronization by PLI, there was no evidence for power law scaling at any frequency. Thus, when we applied the pre-processing and signal analysis methods exactly as reported by Kitzbichler et al. to the same data reported by Shriki et al., we did not reproduce power law scaling of synchronization in empty room MEG recordings.

## Conclusions and Implications

Specifically in relation to Dr Farmer’s comment that highlighted this issue and motivated our reanalysis of these data, we conclude that the methods and results reported by Kitzbichler et al. are not, in fact, compromised by the apparently problematic results reported by Shriki et al. The evidence for power law scaling of synchronization metrics in empty room data provided by Shriki et al. is not reproduced when exactly the same methods described by Kitzbichler et al. are applied to analysis of the empty room data reported by Shriki et al. We therefore consider that the observation of power law scaling in PLI measures on empty room data reported by Shriki et al. [2] does not indicate that the methodology reported by Kitzbichler et al. [1] is “unsound” or “problematic” in the fundamental sense implied by Dr. Farmer’s reading of these two papers. This is because the results reported by Shriki et al. were not based exactly on the methods described by Kitzbichler et al.; when the same empty room data were pre-processed for line noise, and the wavelet-based PLI estimator was used, there was no evidence for power law scaling of empty room MEG data.

Care should be taken when applying the PLI metric to new MEG data whose background noise structure may not be previously well understood. Standard pre-processing steps have to be applied, such as line-noise removal or signal-space separation, but one should be mindful of the fact that all of them also change the covariance structure of the data.

More generally, we conclude from this analysis that it is indeed a useful “sanity check” to test any proposed method for MEG analysis on data recorded from an empty room. Consistency of results between empty room and human recordings may be indicative of a confounding effect of the instrumentation or analysis: in this case, the presence of low amplitude line noise substantially contributed to the existence of power law scaling in the empty room data reported by Shriki et al. [2]. We also conclude that, despite the additional computational cost of wavelet-based metrics of phase synchronization, wavelets may provide a superior basis for estimation of phase relationships in multiscale, non-stationary time series.
